# FAIR environmental and health registry (FAIREHR)- supporting the science to policy interface and life science research, development and innovation

**DOI:** 10.3389/ftox.2023.1116707

**Published:** 2023-06-05

**Authors:** Maryam Zare Jeddi, Karen S. Galea, Susana Viegas, Peter Fantke, Henriqueta Louro, Jan Theunis, Eva Govarts, Sébastien Denys, Clémence Fillol, Loïc Rambaud, Marike Kolossa-Gehring, Tiina Santonen, Hilko van der Voet, Manosij Ghosh, Carla Costa, João Paulo Teixeira, Hans Verhagen, Radu-Corneliu Duca, An Van Nieuwenhuyse, Kate Jones, Craig Sams, Ovnair Sepai, Giovanna Tranfo, Martine Bakker, Nicole Palmen, Jacob van Klaveren, Paul T. J. Scheepers, Alicia Paini, Cristina Canova, Natalie von Goetz, Andromachi Katsonouri, Spyros Karakitsios, Dimosthenis A. Sarigiannis, Jos Bessems, Kyriaki Machera, Stuart Harrad, Nancy B. Hopf

**Affiliations:** ^1^ National Institute for Public Health and the Environment (RIVM), Bilthoven, Netherlands; ^2^ Institute of Occupational Medicine (IOM), Research Avenue North, Riccarton, United Kingdom; ^3^ NOVA National School of Public Health, Public Health Research Centre, Comprehensive Health Research Center, CHRC, NOVA University Lisbon, Lisbon, Portugal; ^4^ Quantitative Sustainability Assessment, Department of Environmental and Resource Engineering, Technical University of Denmark, Kgs. Lyngby, Denmark; ^5^ National Institute of Health Dr. Ricardo Jorge, Department of Human Genetics, Lisbon and ToxOmics - Centre for Toxicogenomics and Human Health, NOVA Medical School, Universidade NOVA de Lisboa, Lisbon, Portugal; ^6^ VITO HEALTH, Flemish Institute for Technological Research (VITO), Mol, Belgium; ^7^ SpF— Santé Publique France, Environmental and Occupational Health Division, Saint-Maurice, France; ^8^ German Environment Agency (UBA), Dessau-Roßlau, Germany; ^9^ Finnish Institute of Occupational Health (FIOH), Helsinki, Finland; ^10^ Wageningen University & Research, Biometris, Wageningen, Netherlands; ^11^ Environment and Health, Department of Public Health and Primary Care, KU Leuven, Leuven, Belgium; ^12^ Department of Environmental Health, National Institute of Health Dr. Ricardo Jorge, Porto, Portugal and EPIUnit—Instituto de Saúde Pública da Universidade do Porto, Porto, Portugal; ^13^ Nutrition Innovation Center for Food and Health (NICHE), University of Ulster, Coleraine, United Kingdom; ^14^ National Food Institute, Technical University of Denmark, Kgs. Lyngby, Denmark; ^15^ Food Safety and Nutrition Consultancy, Zeist, Netherlands; ^16^ Department of Health Protection, Laboratoire National de Santé (LNS), Dudelange, Luxembourg; ^17^ HSE—Health and Safety Executive, Buxton, United Kingdom; ^18^ UK Health Security Agency, Radiation, Chemical and Environmental Hazards Division, Chilton, United Kingdom; ^19^ Department of Occupational and Environmental Medicine, Epidemiology and Hygiene, Italian Institute Against Accidents at Work (INAIL), Monte PorzioCatone(RM), Italy; ^20^ Radboud Institute for Biological and Environmental Sciences, Radboud University, Nijmegen, Netherlands; ^21^ esqLABS GmbH, Saterland, Germany; ^22^ Unit of Biostatistics, Epidemiology and Public Health, Department of Cardio-Thoraco-Vascular Sciences and Public Health, University of Padua, Padova, Italy; ^23^ Federal Office of Public Health, Bern, Switzerland; ^24^ Swiss Federal Institute of Technology (ETH) Zurich, Zurich, Switzerland; ^25^ State General Laboratory, Ministry of Health, Nicosia, Cyprus; ^26^ HERACLES Research Center on the Exposome and Health, Center for Interdisciplinary Research and Innovation, Aristotle University of Thessaloniki, Thessaloniki, Greece; ^27^ Complex Risk and Data Analysis Research Center, University School for Advanced Studies IUSS, Pavia, Italy; ^28^ Laboratory of Pesticides’ Toxicology, Department of Pesticides Control and Phytopharmacy, Benaki Phytopathological Institute, Kifissia, Greece; ^29^ School of Geography, Earth, and Environmental Sciences, University of Birmingham, United Kingdom; ^30^ Centre for Primary Care and Public Health (Unisanté), University of Lausanne, Lausann e, Switzerland

**Keywords:** open science, preregistration, environmental medicine, metadata, exposure science, data-driven decision making, exposure-disease continuum, real-world data

## Abstract

The environmental impact on health is an inevitable by-product of human activity. Environmental health sciences is a multidisciplinary field addressing complex issues on how people are exposed to hazardous chemicals that can potentially affect adversely the health of present and future generations. Exposure sciences and environmental epidemiology are becoming increasingly data-driven and their efficiency and effectiveness can significantly improve by implementing the FAIR (findable, accessible, interoperable, reusable) principles for scientific data management and stewardship. This will enable data integration, interoperability and (re)use while also facilitating the use of new and powerful analytical tools such as artificial intelligence and machine learning in the benefit of public health policy, and research, development and innovation (RDI). Early research planning is critical to ensuring data is FAIR at the outset. This entails a well-informed and planned strategy concerning the identification of appropriate data and metadata to be gathered, along with established procedures for their collection, documentation, and management. Furthermore, suitable approaches must be implemented to evaluate and ensure the quality of the data. Therefore, the ‘Europe Regional Chapter of the International Society of Exposure Science’ (ISES Europe) human biomonitoring working group (ISES Europe HBM WG) proposes the development of a FAIR Environment and health registry (FAIREHR) (hereafter FAIREHR). FAIR Environment and health registry offers preregistration of studies on exposure sciences and environmental epidemiology using HBM (as a starting point) across all areas of environmental and occupational health globally. The registry is proposed to receive a dedicated web-based interface, to be electronically searchable and to be available to all relevant data providers, users and stakeholders. Planned Human biomonitoring studies would ideally be registered before formal recruitment of study participants. The resulting FAIREHR would contain public records of metadata such as study design, data management, an audit trail of major changes to planned methods, details of when the study will be completed, and links to resulting publications and data repositories when provided by the authors. The FAIREHR would function as an integrated platform designed to cater to the needs of scientists, companies, publishers, and policymakers by providing user-friendly features. The implementation of FAIREHR is expected to yield significant benefits in terms of enabling more effective utilization of human biomonitoring (HBM) data.

## 1 Introduction

**FIGURE 1:**
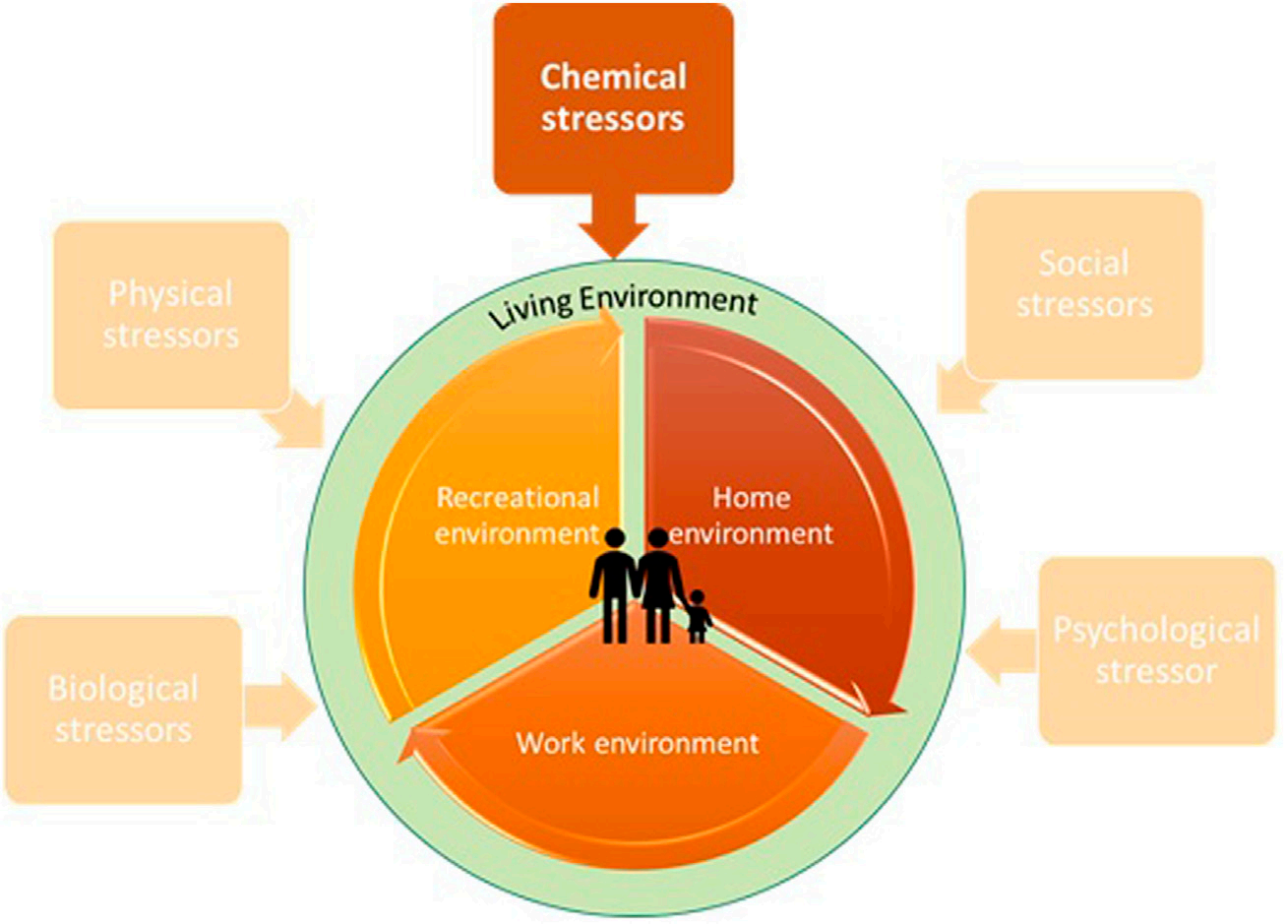
The interface of human health and the environment.

Life is a complex phenomenon that arises from the interplay of multiple factors, including genetics, metabolism, nutrition and the environmental factors. However, the presence of, stressors in our living environment such as chemicals and their mixtures, can potentially impair human life by potentially disrupting physiological functions at the cellular, molecular, or organismal levels (Figure 1) (Naidu et al., 2021). Environmental health span many diverse disciplines including exposure science and environmental epidemiology and plays an important role in addressing pressing and complex issues on how people are exposed to hazardous chemicals and their related health consequences aiming at identifying measures and strategies to reduce exposures and protect human and environmental health (Heacock et al., 2022).

Although development of disease over the course of life is multi-faceted, and disentangling the relationship between environmental contamination, exposure, and disease is complex, advances in data integration, interoperability and (re)use will offer a path forward to explore data across disciplines to reveal new insights.

The “Europe Regional Chapter of the International Society of Exposure Science” (ISES Europe) human biomonitoring working group (ISES Europe HBM WG) recently published their strategic objectives of which one objective was to generate high quality Human biomonitoring (HBM), as a tool in exposure science and environmental epidemiology, by harmonising data life-cycle and implementing FAIR (findable, accessible, interoperable and reusable) guiding principles (Wilkinson et al., 2016; Fantke et al., 2022; Zare Jeddi et al., 2022). The aim is to improve and expedite HBM data integration, interoperability and (re)use in regulatory exposure and risk assessment (as well as risk-benefit assessment), public health policy development, and research, development and Innovation (RDI) in Europe and beyond (Zare Jeddi et al., 2022).

In addition, FAIR data are considered key components of the new EU Industrial Strategy, Chemicals Strategy for Sustainability and Circular Economy Action Plan driven by the EU Green Deal approach, which aims to support substitution and elimination of hazardous chemicals based on Safe and Sustainable by Design approaches and thus enable safe and sustainable innovation (Marx-Stoelting et al., 2023).

HBM means sampling and analysis of biological matrices (e.g., blood, urine, saliva, hair, nails, buccal cells) from individuals or populations. This is to assess internal exposure in an integrated manner (combining all routes and sources of exposure) of xenobiotics by measuring the parent substance, products of biotransformation (e.g., exposure biomarkers such as chemicals themselves and their metabolites) and/or early indicators of effect (effect biomarkers such as micronuclei) (WHO, 2015; Fantke et al., 2020b). Epidemiological studies that measure the strength of associations between internal chemical exposures and human health effects or diseases (e.g., cancer) are also classified as HBM studies (Sexton et al., 2004). Therefore, HBM, in exposure science and environmental epidemiology, provides real world data and evidence addressing pressing and complex issues on how people are exposed to hazardous chemicals and their related health consequences. HBM can improve risk assessment, disease prevention, and life sciences RDI (e.g., integrate data to improve biosensor probes used to detect and quantify pollutants in the environment or humans). At the risk management level, HBM can help in prioritizing exposure mitigation actions, evaluating existing regulatory measures, and assisting in evidence-based policy decision-making for safer chemicals management and public health (Zare Jeddi et al., 2022). Furthermore, well annotated and harmonised HBM data can effectively support the development of an efficient early warning system in Europe, able to capture emerging chemicals of concern and facilitate its widespread deployment. Finally, access to such data can support the implementation of the safe and sustainable by design (SSbD) framework of the European Commission in direct support to the EU Green Deal and the Chemicals Strategy for Sustainability by providing the necessary data infrastructure for developing viable alternatives to hazardous chemicals or chemicals of concern, thereby enabling effective chemical substitution along the lines of the SSbD principles. Secondary harmonised HBM data can also be used in the context of read across methodologies and in silico predictive models for toxicology to predict physico-chemical and ADME properties, toxicological effects and internal exposure at early stages in the chemical innovation process (Cronin et al., 2023).

An additional benefit from well annotated and harmonised HBM data is their potential use for exposure reconstruction, i.e., the assimilation of human biomonitoring data and their translation into intake distribution. This is a computational inversion problem, where the objective is to identify the specific input distributions that best explain the observed outputs (measured HBM data) while minimizing the residual error. A major advantage of this method is that the intake estimates can be directly assessed against existing external reference levels (RfD, TDI, RfC, etc.), facilitating in a transparent manner the risk assessment process. The above method has been effectively applied for the risk assessment of bisphenols (Sarigiannis et al., 2016; Sarigiannis et al., 2019) and ortho-toloudine (Huuskonen et al., 2022).

The ISES Europe HBM WG proposes to optimise future HBM, as an important tool in environmental health studies providing real world evidence, by increasing its harmonization in the various parts of the study set-up and at the same time improving findability, comparability, integrability, interoperability, and reusability of HBM data for societal benefits (Zare Jeddi et al., 2021). The ISES Europe HBM WG might serve as anchor point for coordinating these activities.

## 2 Challenges

Considering the ongoing rise in scholarly output (Bornmann et al., 2021; Bornmann and Haunschild, 2022), the changing market dynamics driven by Open Science policies, and the constantly changing stakeholder expectations (National Academies of Sciences Engineering and Medicine, 2018; Chakravorty et al., 2022), it is more important than ever to move towards openness, transparency, and reproducibility of HBM research and its outputs. The aim is to increase the quality and impact of knowledge and research, among other things, by making it findable as easily as possible and accessable when provided.

Often finding existing and ongoing research projects is time consuming and this lead to the main challenge that data are not easily findable and retrievable in different sources. When such sources are accessed and studied, found and accessed the comparability of existing HBM data is often limited due to specific challenges, for example, differences in the design of different studies and the quality criteria of the employed analytical methods. Moreover, integrating HBM data with other physicochemical, human toxicity, demographic, environmental, or food data remains a challenge due to often lack of metadata and information needed to support interoperability and provide context. Interoperability can be seriously challenged by the lack of domain specific metadata (Daniel et al., 2022; Emara et al., 2022; Kosnik et al., 2022).

Some of challenges hindering data integration and reuse are listed below:

- Insufficient metadata collection (Metadata deficiencies);
- Data type misalignment;
- Lack of harmonised terminologies and use of ontologies (e.g., dictionaries for common variables across HBM studies to facilitate combining data);
- Inadequacy of existing ontologies e.g., chemicals not explicitly identified in ontologies, particularly for mixtures;
- Data from diverse sources are not standardized in terms of how contaminants are described, how samples are characterized, or when and how they are taken;
- Metadata not interoperable due to different formats;
- Differences in units of measurement (not converted or reported units);
- Differences in terminology, and instruments across labs resulting in difficulties in integrating data across different populations;
- Inconsistent processes, methods, and software used across labs;
- Lack of lab metadata (e.g., lack of critical metadata relating to environmental conditions and samples preparation);
- Poor replication rate;
- Selective reporting and publication bias, particularly the prioritization of publishing positive over negative results.

Some researchers may not be aware of existing ontologies (e.g., FoodOn, UMLS), how to find or select the most appropriate ontology, or how to use them. Each area of science may have their own vocabularies, and more specific subdisciplines may lack or have incomplete vocabularies, further complicating their use. Even discrete communities within the same scientific discipline may format and describe data differently, pointing to the need for standardizing these processes to integrate data streams.

Overall, the lack of accessible metadata on projects leads to unawareness of a complete set of HBM data and missed opportunities. As a result, useful information is missed, resources are potentially wasted (or certainly not used to maximum potential), the community is not able to build on previous knowledge and HBM cannot reach its full potential as a policy-support tool (Burgelman et al., 2019). There are currently only few centralized international data repositories or access portals for collection and storage of HBM (meta)data (see Annex 1 for a listing of these). Data repositories have in general varying metadata sets, and have inconsistent protocols for collecting and storing data. Data in different repositories often lack essential metadata, often has consistency errors, missing data, and inconsistent use of field names across datasets. In addition, the quality assessment and curation efforts are manual and inconsistent as existing repositories lacked clear procedures for receiving, for example, environmental contaminant data needed for reuse (Doiron et al., 2013; Fantke et al., 2020a; Aurisano and Fantke, 2022). Efforts have been made mainly in a cooperation between IPCHEM^1^ and HBM4EU^2^ to create harmonised reporting formats and datasets, for instance, via the HBM4EU Europe-wide aligned studies (Gilles et al., 2021; Gilles et al., 2022; Govarts et al., 2023). Furthermore, a harmonized meta (data) catalogue is expected as an outcome of the European Human Exposome Network ^3^ (EHEN) which aims to harmonize (meta)data across its 9 projects. This catalogue is available through the EHEN Molgenis data platform ^4^ and contains metadata on cohorts/data sources, the variables they collect, and/or harmonization efforts to enable integrated reuse of their valuable data.

Despite continued efforts towards harmonization and interoperability of (meta)data by ongoing European initiatives such as the European Partnership for the Assessment of Risks from Chemicals (PARC), and EHEN, the process of implementing the FAIR (findable, accessible, interoperable, reusable) guiding principles for scientific data management and stewardship across the global scale remains a challenge (Wilkinson et al., 2016; Govarts et al., 2022). Furthermore, the EU and national ethical and legal obligations often restrict data sharing, grouped analysis, and data deposition.

Today, information about HBM study metadata is accessible only after publication. Large-scale initiatives, such as PARC, HBM4EU, the German Environmental Specimen Bank^5^, the Canadian Health Measures Survey (CHMS)^6^, and NHANES^7^ have their own website. The project related websites provide additional information about the ongoing project, which is not always the case for smaller sized projects. In addition, if projects/websites are available, the scientific community or other interested stakeholders are not always aware of these (not findable).

Research planning is critical to setting data up to be FAIR at the outset. This includes an informed strategy about what data and metadata to collect, how it will be collected and described, and approaches for assessing its quality and its management (Heacock et al., 2022). Development of a global registry would allow collection of metadata (decisions around study design, methods, and analysis) of all planned HBM studies.

A global registry is clearly different from a data repository as no measurement data will be stored. This distinction is important as this registry could be consulted freely to understand what studies are planned, underway, or completed without data ownership and information privacy concerns.

Preregistration has been a commonly used practice in research for some time in several different research disciplines, such as psychology (Nosek et al., 2018), biomedical research (Serghiou et al., 2023), applied research (Evans et al., 2023), and animal research (van der Naald et al., 2021), among others. A registry specifically designed for collation of HBM studies performed worldwide prior to data being collected/accessed (i.e., preregistration of HBM studies) is currently lacking. Non-exhaustive list of templates and platforms for (pre)registration of research studies are summarized in Annex 2, Table 1. Whilst the focus in the past has been on registering randomised controlled trials (RCTs) (Serghiou et al., 2023), the current need is to register the ever-growing number of HBM observational studies (cohort, cross-sectional, etc.) and increase the validity and transparency of research in this field. EHS can adopt and implement preregistration successfully by learning from existing registries and provide means, motive, and opportunity for rigor and robustness of research practices via preregistration.

## 3 Concept and scope

The ISES Europe HBM WG’s goal is to develop a global preregistration (i.e., specifying the research plan in advance of the study and submitting it to a registry platform) of environmental health studies, focussing initially on those in the HBM domain, in line with Open Science practices^8^ (Zare Jeddi et al., 2021) and subjected to peer-review. The proposed global registry is called FAIR environmental and health registry (FAIREHR)*,* which aims to promote and facilitate FAIR by design studies. The FAIR data principles apply not only to data, but also to metadata, supporting infrastructure (e.g., search engines) and other research outputs. At the metadata level, findability and accessibility requirements must be addressed, while interoperability and reuse require more efforts at the data level (Sinaci et al., 2020). This global registry would ensure that research outputs (often funded with public budget) have a larger impact in the long-term. Preregistration of HBM studies enables researchers to specify and share the details of their research, decisions around study design, data management plans (DMP), methods, and analysis in a public registry before conducting the study. Therefore, extra effort and additional steps will not be required to creating DMP when using FAIREHR. DMP will be generated for each project outlining what research data were collected, how they were collected and what you will do with your data during and after your research.

**FIGURE 2:**
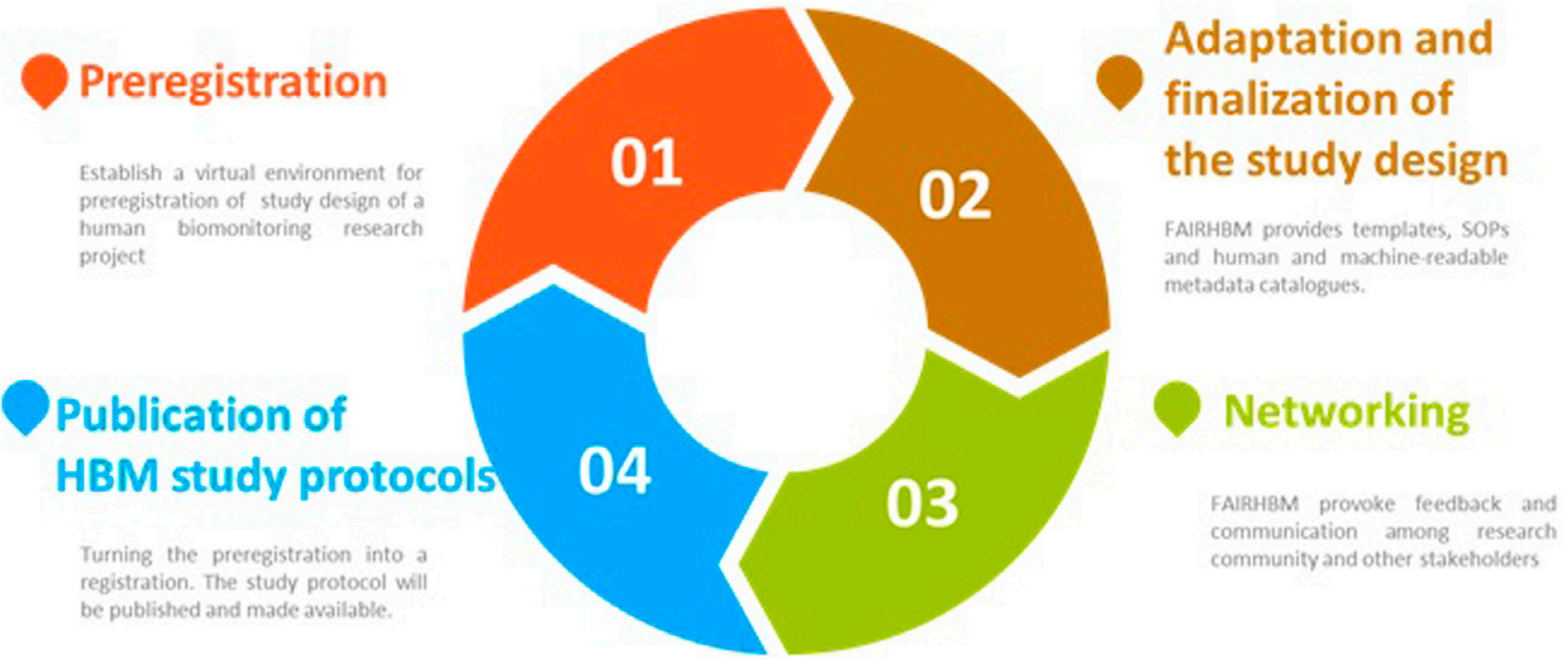
The features of FAIREHR as a “global registry.”

Utilising the registry will also stimulate communication and interaction among HBM communities and can lead to potential adjustments and/or alignments with other HBM study designs upfront resulting in more harmonized and robust study designs worldwide. The general features offered by FAIREHR is shown in Figure 2.

**FIGURE 3:**
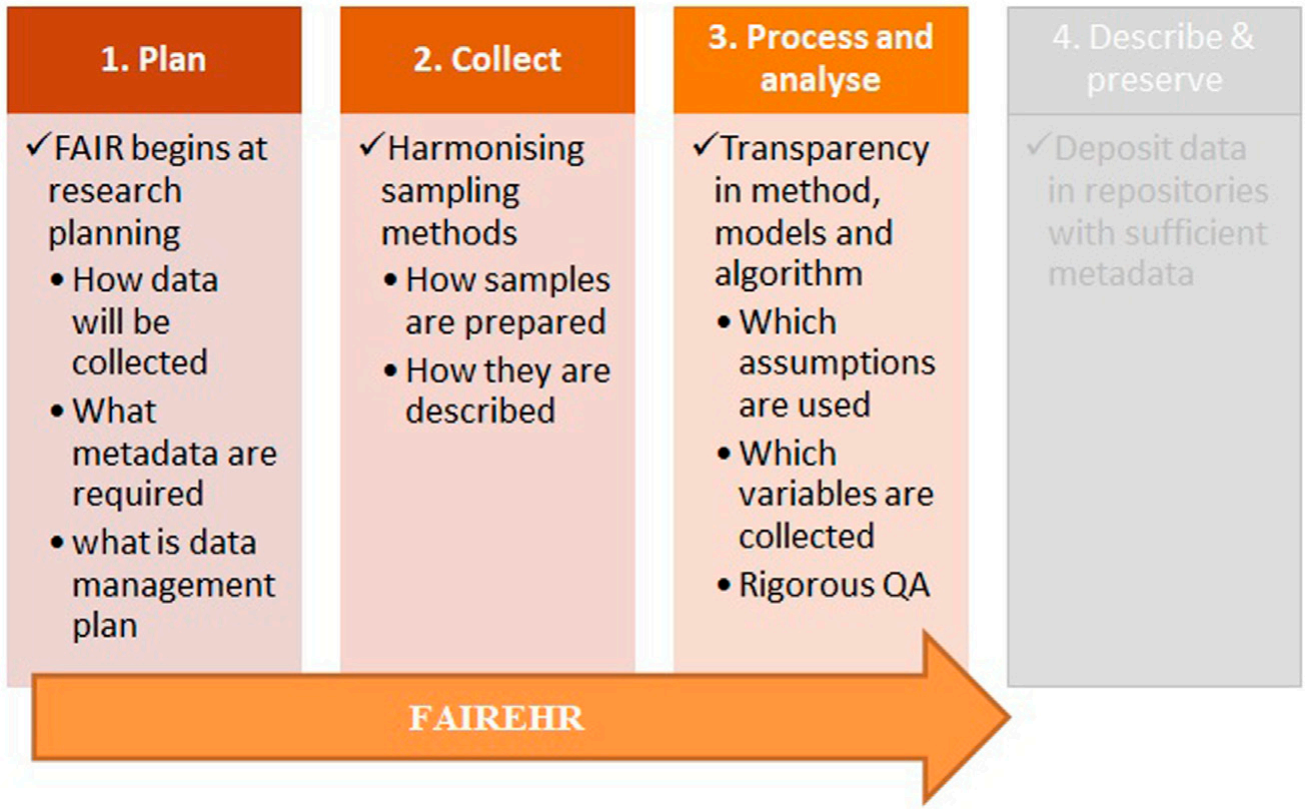
Preregistration of environmental health studies in FAIREHR will facilitate FAIR data principles implementation which begins with research planning and extend through the data lifecycle.

Implementation of FAIR principles throughout the data lifecycle (FAIRification) is the main goal of FAIREHR to make sure that a research project will deliver reusable data. For data to be usable across disciplines and reusable for other purposes, they must be clearly described. Key information needs to be reported on data collection methods, lab protocols, and data formats as robust metadata are fundamental for data interpretation, sharing, integration, interoperability and reuse. Furthermore, descriptive human and machine-readable metadata are an essential component for machine discoverability of data sets and services for efficient public health policy and RDI (Heacock et al., 2022). Therefore, implementation of FAIR data principles begins with research planning and extend through the data lifecycle. This is the core concept of FAIREHR (Figure 3) to stimulate researchers to consider the value of FAIR principles already in the planning phase. Additionally, the best point of action for quality control is at the point of data collection; therefore, we argue that in the absence of a review-based registry (such as FAIREHR) the integration challenges and quality issues usually emerge late at the time of analysis and reuse.

Finally, the objective of FAIREHR is to provide researchers with equitable access to contemporary templates, standard operating procedures (SOPs), and protocols, enabling them to enhance their study designs and produce comparable, reproducible, and integrable research outcomes. We do not support rejection of preregistrations and in special cases preregistrations will only be rejected if the researchers do not wish to utilize the templates and protocols provided by FAIREHR for a specific research topic with available harmonized template and protocols. We aim to develop FAIREHR to be user-friendly for both users and reviewers such as simple and clear user interface (straightforward instructions and design), clear and easy to follow navigation with labels and easy-to-understand icons (tooltips, explanations of terms, and other helpful information), data entry interface with dropdown menus, radio buttons, and other input controls following structured questions, (yes/no and multiple-choice formats), robust search and filtering capabilities, online help desk, and finally, strong security features to protect user data and prevent unauthorized access.

### 3.1 Technical setup of FAIREHR

**FIGURE 4:**
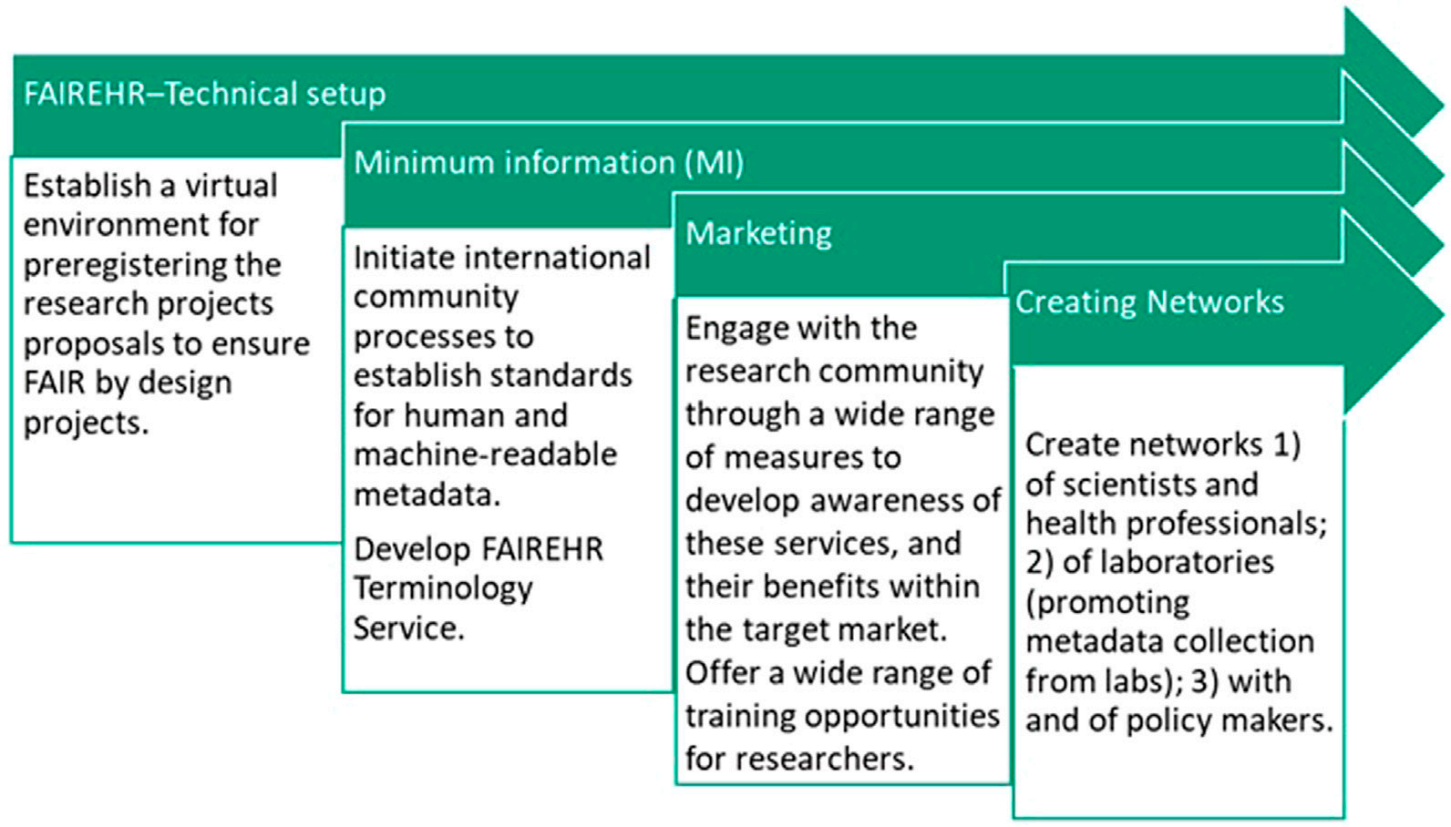
Description of a step-wise implementation of the FAIREHR.

There are varying levels of technical setup required for the development of FAIREHR (Figure 4). Successful deployment of FAIR will require a standardised information architecture and environmental and occupational health research community should reach a robust consensus on balanced minimum information guidelines to ensure necessary metadata are collected without placing undue burden on users, and on development, and implementation of relevant semantic standards, such as ontologies or hierarchical vocabularies to capture specific types of data (Mattingly et al., 2016). To do so, FAIREHR will:

1) adopt uniform resource identifiers (URIs) or persistent identifiers (PIDs) as part of its data lifecycle management strategy. The use of URIs or PIDs is recognised as key performance indicators of the FAIRification process of data lifecycle (Collins et al., 2018; Wise et al., 2019). This is consistent with the Findability guidelines (i.e., F1), which emphasizes the importance of making data Findable by assigning them with globally unique and persistent identifiers.
2) promote alignment of the studies by making available SOPs as well as harmonised and agreed open access templates for each step of any type of HBM study as a starting point. This will include the specifics of research design and analysis plans. This process ensures that relevant metadata are collected and data entries are uniform. This is consistent with the Findability guidelines (i.e., F2) as well as defined by reusability guidelines (i.e., R1), which emphasizes that data should be described with rich metadata.
3) initiate establishment of balanced minimum information guidelines in collaboration with broad community of stakeholders including researchers and clinicians (data generators and data users), publishers, industry and government agencies. This is consistent with the findability guidelines and useability guidelines to optimise the reuse of data. To achieve this, metadata and data should be well-described so that they can be replicated and/or combined in different settings.
4) accommodate relevant ontologies. This aligns with I1 and I2 to insure Interoperability of data.

Data and metadata will be described using normative and community recognized specifications, harmonized codebooks for biomarkers, vocabularies, related ontologies, and standards that determine the precise meaning of concepts and qualities that the data represent. Users can browse or search the ontologies and use links on the metadata to find related information. Ontologies can facilitate data integration by standardizing the vocabulary used to describe different entities and relationships between them. For example, in the case where clinical information is captured, systems need to “know” that phenotypic terms such as “microcephaly,” “nanocephaly,” “small head,” among others, have some equivalence, and that requires a coordinated use of ontologies.

The scope of FAIREHR includes HBM studies of any design and for any population. Examples of designs include: cross-sectional studies, case-control studies, prospective designs, and retrospective studies. Cross-sectional designs may aim at comparing biomarkers across populations using well-standardized methods of sample collection and analysis (Santonen et al., 2019; Scheepers et al., 2021; Silva et al., 2021; Jones et al., 2022). HBM studies may follow populations prospectively such as in birth cohorts (Andersen et al., 2016; Nakayama et al., 2019) or follow populations impacted by chemical incidents and disasters (Scheepers and Smolders, 2014a). HBM studies at varying scales, including large-scale multicentre, multinational surveys, or small-scale studies will be included as long as HBM is performed according to well-established protocols (Scheepers et al., 2011; Scheepers et al., 2014b).

The FAIREHR would have a pool of experts registered as reviewers so that the process is not too onerous on a limited group of individuals. The initial set of reviewers will comprise our scientific advisory board, who have been actively involved in the development of this platform from its inception and possess comprehensive knowledge of all facets of FAIREHR. The FAIREHR employs structured questions including yes/no and multiple-choice formats. This should reduce the use of open-ended queries and facilitate a straightforward process for both registrant and reviewers. Moreover, a set of review criteria should guide reviewers in their evaluation of registrations. Each registry record would be reviewed by experts from a related field without competing interests. Consequently, the serving reviewers must disclose any potential conflicts of interest.

The FAIREHR will most likely be hosted by ISES or a non-governmental/non-for-profit expert organization. Any registered study will be subject to an embargo period before being published online. For instance, the *Journal of Environmental Exposure Assessment* (JEEA)^9^ has agreed to be connected to the FAIREHR to publish study protocols. Moreover, JEEA has agreed to include the FAIREHR recommendations in its Guide for Authors for those wishing to publish protocols of HBM studies. Embargo period will be clearly communicated with registrants. FAIREHR will also engage with repositories to ensure that the necessary metadata requirement is met to enable interoperability and reuse.

These protocol articles would include study background and design with descriptions of recruitment, sampling, chemical analysis (including acceptance criteria for the analysis), and statistical analysis as well as the data management plan. After completion of the study, ideally the data would be fed into a database for HBM data that then includes the link back to the FAIREHR, for retrieval of the full metadata. FAIREHR would provide an environment for HBM researchers to initiate an active HBM science network. The FAIREHR will enable researchers to consult with experts in the field to obtain insights in topics relevant for their research question. In addition, human and machine-readable metadata will enable automatic discovery of datasets and innovations to leverage science-based decision-making processes (at technical and policy levels) for public health.

Technical steps such as integrating FAIREHR with other tools and platforms would be a valuable way to expand its functionality and enhance its usefulness long term, but possible legal issues will need to be addressed before proceeding with any integration. Overall, as FAIREHR continues to develop and mature, exploring integration with other tools and platforms can be a strategic goal to pursue.

## 4 Expected impacts and benefits

Publicly available metadata enhances the visibility of existing interoperable data facilitating communication and collaboration among researchers, for example, those engaged in risk assessment and exposure science. Preregistration of research studies aids, scientists, regulators, policymakers (at various levels such as EU, national, regional), life sciences RDI organisations, publishers, and research funding bodies to track and identify planned and ongoing, as well as completed, studies. For example, it would save time by quickly finding which HBM studies an organisation has, which departments and people can arrange access to datasets of interest, or which data licenses and request procedures are required. In particular, an overview of on-going research can provide valuable insights into existing gaps and facilitate prioritization of future research endeavors and optimize resource allocation.

In situations where data are restricted for security purposes (e.g., personally identifiable information) the metadata would include information about how the data can be accessed and what permissions are needed so researchers may still be able to share some human data through restricted and secure access.

Preregistration helps raise awareness of non-significant findings, potential low statistical power in studies, publication bias, and thus helps researchers learn for the lessons gained by others by making all planned studies discoverable whether or not they are ultimately published (Nosek et al., 2019; Rubin, 2019; Paul et al., 2021).

The FAIREHR will serve the research community from concept to project completion (registering studies that are planned, ongoing, and completed) for generating reusable high-quality metadata and ensuring visibility throughout the research project life-cycle. Preregistration of HBM studies and producing more structured human and machine-readable metadata with FAIREHR will:

1) Maximize resource efficiency by increasing the interaction between investigators to enable harmonization of study designs before measurements commence, thereby reducing wasted effort and research funds;
2) Provide a harmonized format for study design to facilitate comparability of research results;
3) Ensure generation of high-quality metadata by ensuring stakeholders are aware of which standards and policies exist and help foster their adoption;
4) Increase transparency in knowledge creation;
5) Encourage dialogues among scientific communities from different geographical locations that will lead to harmonized research approaches and thus, comparability of multi-country data.
6) Increase findability of measurement data by linking every registered study to prospective data repositories;
7) Increase the accessibility of study information (protocols and HBM metadata) and of eventual measurement data;
8) Facilitate the findability of HBM research and increase awareness of previous projects.

Therefore, full deployment of the FAIREHR could lead to higher research productivity, impactful research, new insights and innovations, as well as improved reproducibility and trust in science.

### 4.1 Expected science to policy impact

**FIGURE 5:**
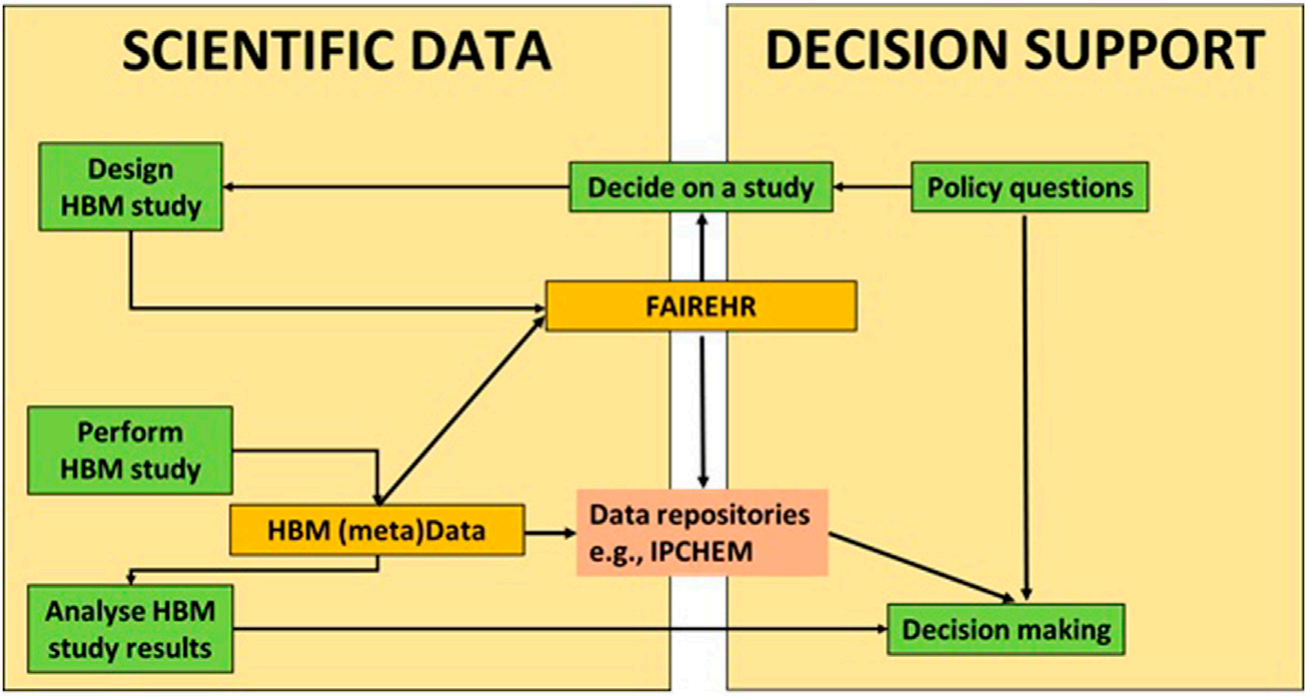
The proposed FAIREHM platform and improved organisation of HBM data and metadata (orange/red boxes) will support the interaction between the common steps (green boxes) in obtaining scientific data and decision making. Green: Actions by policymakers or researchers, Orange: Results from these actions, and the main elements for the proposed new system. Red: Existing data repositories.

The FAIREHR will directly enable the interface between science and policy (regulators and policymakers) as illustrated in Figure 5. The proposed FAIREHM platform and improved organisation of HBM data and metadata (depicted by orange and red boxes, respectively) are expected to facilitate the interplay between the various stages involved in generating scientific data and arriving at informed decision making (highlighted by green boxes). It is important to highlight that science possesses the capability to investigate queries posed by policymakers, while decisions will invariably be made by policymakers. Maintaining a clear boundary between the domains of science and policy is crucial. The FAIREHR would enable a science-policy interface where policymakers have access to on-going, completed and planned HBM studies and can articulate their needs for data and future research. Meanwhile, researchers will have the opportunity to communicate directly with policymakers but also within a global HBM network. Additionally, FAIREHR would strengthen the transparency of environment and health research studies in line with recommendations made in the Regulation (EU) 2019/1381 of the European Parliament and of the Council of 20 20 June19^10^ on the transparency and sustainability of the EU risk assessment for food and use features from the European Food Safety Authority (EFSA) toolkit^11^ geared towards enhancing transparency.

The FAIREHR science and policy interface benefits are:

1) Facilitate easier/more efficient identification of available HBM projects that could be sources of information fit for the purpose of regulatory risk assessment (and risk-benefit assessment), thus addressing policy questions in a timely manner with least effort;
2) Assure transparency and reduce bias e.g., publication bias;
3) Promote dialogues among scientists, policymakers and practitioners, entrepreneurs, and community members during the research process such that they can voice their concerns, needs and aspirations;
4) Create possibilities for regulators to communicate their needs regarding HBM data for specific substances, substance groups and/or target populations, leading to the design of fit for purpose research based on policy questions and public concern;
5) Provide an environment for HBM researchers to network and for regulators to accelerate policy progress through establishing connections, starting conversations, engaging in discussion and collaboration.

### 4.2 Expected business impact

Four short-term time and cost savings envisaged are:

1) excellent findability for relevant research projects;
2) quick access to the data at scale;
3) standardised and high-quality data ready for selection in analytical processes such as machine learning;
4) support to chemical innovation along the lines of the SSbD framework; a key information and data resource for small and medium enterprises in the chemical sector.

In the longer term, lower costs to society will accrue, owing to decreases in the burden of disease. HBM provides insight into the exposure-disease continuum, and thus, enable the construction of precise, comprehensive and translational study of the effects of the environment on humans. For example, HBM can be used to stratify patient populations to identify patients who might benefit from a clinical trial, alert of safety issues that can thus be avoided. Furthermore, improved search results for HBM studies that show effects from an unhealthy lifestyle and living environment would be provided with direct access to the FAIREHR as well as a demonstrable increase in productivity of the R&D pipeline. EHS data integration and interoperability can identify innovative solutions to environmental health problems and open new opportunities to maximize previous and future research investment while advancing science more rapidly and in areas not previously possible.

## 5 A stepwise approach to FAIREHR’s implementation

The challenges towards creating FAIREHR are substantial and we, therefore, propose a stepwise approach for its development. As FAIREHR evolves, it will attain varying levels of maturity over time, and we will utilize solutions to expedite the FAIRification process.

The ISES Europe HBM WG on FAIREHR (the “FAIREHR team”) foresees close collaboration with the European initiative PARC. One aspect covered by PARC aims to enhance the EU experimental capacities, increase reliability/quality of newly generated information and will work on FAIRification of HBM datasets (amongst many other activities). Within PARC there is a 3.5 years internal project on FAIR and sustainable storage of HBM datasets. It will systematically address all 15 FAIR principles, and include, e.g., development of FAIR metadata, metadata schemas and templates, vocabularies and ontologies, as well as (sustainable) FAIR data repositories. This highlights, the complementarity and emphasizes the need for close collaboration between PARC and the FAIREHR team. This collaboration will help define the FAIREHR required dataset. The FAIREHR will capture key attributes of HBM study designs carried out worldwide. These submitted registration forms would be checked for coherence, completeness, and clarity before being made publicly available. The resulting FAIREHR as a meta-database would contain public records of metadata such as study design, an audit trail of major changes to planned methods, HBM data format and structure form each study, details of when the study will be completed, and links to resulting publications and where data are stored (e.g., data repositories) when provided by the authors thus FAIREHR will not contain measurement data itself. Such a meta-database would thus act as an important resource in the science-stakeholder interface for raising awareness of upcoming studies/data in the field of environmental health.

These metadata would provide basis to improve future HBM studies. The FAIREHR would be an integrated service for scientists, life sciences companies, publishers and policymakers with user-friendly features and consequently, science will serve society in a more effective manner.

Funding is required to develop and build the FAIREHR and science governance to ensure its sustainability. A governance model is essential for maintaining the platform, sustaining community efforts in keeping with scientific and technical advances, and championing public availability of standards for EHS data to ensure continued relevancy. Support from international projects and consortia could potentially provide seed funding for development of FAIREHR. The registry to be free of charge for individual researchers however dedicated funding mechanisms for its sustainability is required (e.g., business to business (B2B) subscription models or advertisements may be options as a revenue model for part of FAIREHR).

Effective and broadly used registry require a high level of scholarship and community involvement, result in major capacity building impacts on research, and are increasingly recognized for their integral role in data analysis and integration.

## 6 Outlook and next steps

The FAIREHR is expected to serve major challenges in implementing a successful “big data” strategy, namely, the quality and completeness of study design and metadata, as well as the time needed to interconvert data from a variety of sources. The FAIREHR would be an integrated user-friendly service for scientists, life sciences companies, publishers, and policymakers. An important goal to be accomplished is that science will serve society in a more effective manner.

## Data Availability

The original contributions presented in the study are included in the article/Supplementary Materials, further inquiries can be directed to the corresponding author.

## Footnotes

1
https://ipchem.jrc.ec.europa.eu/

2
https://www.hbm4eu.eu/

3
https://lifecycle-project.eu/for-scientists/the-eu-child-cohort-network/

4
https://data-catalogue.molgeniscloud.org/catalogue/catalogue/#/networks-catalogue

5
www.umweltprobenbank

6
https://www.statcan.gc.ca/en/survey/household/5071

7
https://www.cdc.gov/nchs/nhanes/index.htm

8
https://research-and-innovation.ec.europa.eu/strategy/str%5dategy-2020-2024/our-digital-future/open-science_en

9
https://www.oaepublish.com/jeea

10
https://eur-lex.europa.eu/legal-content/EN/TXT/?uri=CELEX%3A32019R1381

11
https://www.efsa.europa.eu/en/applications/toolkit#connect-efsa
