# Supplementary material for: FAIR environmental and health registry (FAIREHR)- supporting the science to policy interface and life science research, development and innovation
Source: Front Toxicol. 2023 Jun 5;5:1116707. doi: 10.3389/ftox.2023.1116707 (PMC10278765; doi:10.3389/ftox.2023.1116707)
Supplement: Supplementary file 1 [file DataSheet1.docx]

**Supplementary Material**

**Annex 1**

The ISES Europe HBM WG recently published their strategic objectives of which one objective was to generate high quality HBM metadata by harmonising existing and future HBM research life-cycle data (Fantke et al. 2022; Zare Jeddi et al. 2022). The intention is to improve and expediate the (re)use of HBM data in regulatory exposure assessment, public health policy development, and exposure science R&D in Europe and beyond (Zare Jeddi et al. 2022). There are currently only few centralized international data repositories or access portals for collection and storage of HBM (meta)data. For instance,

- IPCHEM^^[[1]](#footnote-1)^^ (the European Commission’s Information Platform for Chemical Monitoring) is a publicly available repository for chemical occurrence data including HBM data (as well as environmental, indoor air and food monitoring data). IPCHEM currently includes metadata for more than 150 HBM studies, aggregated HBM measurement data, and few anonymized individual data are stored. In addition, IPCHEM is an access portal to many other HBM data stored in national data repositories e.g. recommended by OECD for its Member Countries.. Aggregated and anonymised data are usually publicly available while pseudonymised individual level data coming in will have strictly restricted access for data protection reasons in the future.
- “[OccupationalCohorts.net](https://occupationalcohorts.net/)” results from two European initiatives, viz the Cost Action [OMEGA-NET](https://omeganetcohorts.eu/) and H2020 project [EPHOR](https://www.ephor-project.eu/) (Pronk et al. 2022) (Kogevinas et al. 2020). This inventory includes information from >100 cohorts and aims to include all active cohorts in Europe (and globally), which can be used to explore occupation, work related exposures, employment and health relationships.
- Project-specific repositories are often developed within various large projects. These are mainly not publicly available for data ‘producers’ to deposit datasets in the repository nor for data ‘(re)users’.
- the Personal Exposure and Health (PEH) data platform^[[2]](#footnote-2)^ (hosted by VITO - Flemish Institute for Technological Research, Belgium) stores pseudonymized individual HBM data from the HBM4EU Aligned Studies (Gilles et al. 2021; Gilles et al. 2022; Govarts et al. 2023), the HBM4EU MOM-study, and in future, data generated in the European Partnership for the Assessment of Risks from Chemicals (PARC). Recently, a Protocol has been drafted and signed by the HBM4EU partners to make the pseudonymized individual HBM data available for reuse under strict conditions in compliance with the EU’s General Data Protection Regulation. New parties that were not partners of the HBM4EU project can sign the Protocol and request access to the data in the PEH platform and/or store new data.
- the National Report on Human Exposure to Environmental Chemicals established by the US Centers for Disease Control and Prevention (CDC)^[[3]](#footnote-3)^ stores data from the CDC’s National Health and Nutrition Examination Survey (NHANES).
- National initiatives e.g. the inventory of Swiss HBM samples and data^[[4]](#footnote-4)^ by the Swiss Biobanking Platform (SBP Next catalogue).

The European HBM dashboard^[[5]](#footnote-5)^, which is another outcome of HBM4EU and will be further maintained in PARC. This dashboard presents aggregated HBM data from the HBM studies but is not meant to be a data repository for direct storage or retrieval of data.

**Annex 2**

**Table 1**. Non-exhaustive list of existing registry templates and platforms in the public domain

| **Platform or registry template** | **Webpage** | **Purpose** | **Discipline/Area** | **Specific for HBM?** |
| --- | --- | --- | --- | --- |
| Occupational cohorts net | <https://occupationalcohorts.net/inventory/> | Explore occupation, work related exposures, employment, and health relationships | Registry of Occupational cohorts | Some extent, i.e., it is not designed for HBM studies for occupational studies and nor for the general population |
| Open Science Framework (OFS) Pre-registration | <https://osf.io/registries> | Multiple templates for preregistration wide range of studies | Any | No |
| Research Registry | <https://www.researchregistry.com/> | Templates for preregistration of systematic reviews and research studies (human participants) | Mainly medical/surgical research, social Science, no registry on Environmental pollutions | No |
| INPLASY | <https://inplasy.com/> | International Platform of Registered Systematic Review and Meta-analysis Protocols | Any | No |
| AsPredicted | <https://aspredicted.org/> | Standardised pre- registration template which provides a simple form for preregistration as nine simple questions about the research design and analyses | Any | No |
| Prospero | <https://www.crd.york.ac.uk/prospero/> | Study protocol registrations for systematic reviews with a health-related outcome | Health and Social Care, Welfare, Public health, Education, Crime, Justice, and International Development | No |
| [International Standard](https://www.isrctn.com/) [Randomised Controlled](https://www.isrctn.com/) [Trials Number (ISRCTN)](https://www.isrctn.com/) [Registry](https://www.isrctn.com/) | [www.isrctn.com](http://www.isrctn.com) | Primary clinical trial registry recognised by WHO and ICMJE | Any clinical research study | No |
| American Economic Association Registry for Randomized Controlled Trials (AEA RCT) | <https://www.socialscienceregistry.org/> | Randomized Controlled Trials Registry in economics, political science, and social science from anywhere in the world. | Economics, Political Science, and other Social Sciences | No |
| Registry for International Development Impact Evaluations (RIDIE) | <https://ridie.3ieimpact.org/> | Prospective registry of impact evaluations for development policies and programmes in low- and middle-income countries | Social Sciences | No |
| Evidence in Governance and Politics (EGAP) | <https://egap.org/registry/> | Registration for experiments and observational studies | Governance and Politics | No |
| Preclinicaltrials | <https://preclinicaltrials.eu/> | A comprehensive listing of preclinical animal study protocols | Animal studies | No |
| animalstudyregistry.org | <https://www.animalstudyregistry.org/asr_web/index.action> | online registry for scientific studies involving animals conducted around the world | Animal studies | No |
| PreReg in Psychology | <https://prereg-psych.org/index.php/rrp> | A long-running discipline-specific registry provided by the Leibniz Institute for Psychology (ZPID). | Psychology | No |
| ClinicalTrials.gov | <https://clinicaltrials.gov/> | privately and publicly funded clinical studies conducted around the world | Clinical trial registrations | few extend |
| International Clinical Trials Registry Platform (ICTRP) | <https://www.who.int/clinical-trials-registry-platform/network/primary-registries> | WHO Registry Network | Clinical trial registrations |  |

1. <https://ipchem.jrc.ec.europa.eu/> [↑](#footnote-ref-1)
2. <https://hbm.vito.be/peh-data-platform> [↑](#footnote-ref-2)
3. <https://www.cdc.gov/exposurereport/data_tables.html> [↑](#footnote-ref-3)
4. <https://swissbiobanking.ch/visibility/> [↑](#footnote-ref-4)
5. <https://hbm.vito.be/eu-hbm-dashboard> [↑](#footnote-ref-5)
